# The Influence of Protein Components on Quinoa Protein–Xanthan Gum Complex Gels at Different pH Levels

**DOI:** 10.3390/gels10120840

**Published:** 2024-12-19

**Authors:** Xinxia Zhang, Yafeng Ding, Jiangtao Zhou, Qianqian Xu, Ting Li, Li Wang

**Affiliations:** 1 State Key Laboratory of Food Science and Resources, Jiangnan University, Lihu Road 1800, Wuxi 214122, China; 8202108006@jiangnan.edu.cn (X.Z.);; 2 Food Science and Technology, Jiangnan University, Lihu Road 1800, Wuxi 214122, Chinaxuqianqian1119@126.com (Q.X.); 3 Jiangsu Provincial Engineering Research Center for Bioactive Product Processing, Jiangnan University, Lihu Road 1800, Wuxi 214122, China; 4 Department of Health Sciences and Technology, ETH Zurich, 8092 Zurich, Switzerland

**Keywords:** quinoa protein, heat-induced gelation, rheological behavior

## Abstract

The study aimed to prepare complex gels of sonicated quinoa protein (QP) and polysaccharides, comparing the effects of different protein components and pH on gel properties. FTIR analysis demonstrated that the β-structure in protein at pH 7.0 was enhanced by ultrasonic treatment, which could promote the formation of a gel network. Moreover, XG-AG (gel prepared by xanthan gum and albumin) and XG-GG (gel prepared by xanthan gum and globulin) exhibited higher levels of disulfide bonds and free sulfhydryl groups in the gel, requiring more energy to break the intermolecular sulfide bonds during heating. Under the same heating conditions, the rheological properties and gel strength of XG-UQPG (gel prepared by xanthan gum and ultrasonically treated QP) were superior to those of XG-UGG (gel prepared by xanthan gum and ultrasonically treated globulin) and XG-UAG (gel prepared by xanthan gum and ultrasonically treated albumin). Additionally, XG-UGG (pH 7.0) demonstrated the highest water holding capacity (WHC) and oil holding capacity (OHC). This was attributed to the disulfide bonds created in the proteins by the ultrasound treatment, encouraging them to interact to form more uniform holes in gel that can hold more water/oil molecules. Conversely, at pH 4.5, the WHCs of the gels were reduced due to the presence of rougher protein structures. These findings shed light on the impact of protein composition on gel properties and offer insights into enhancing the quality of quinoa protein gel.

## 1. Introduction

The study of thermally induced phyto-protein gels has attracted attention because of their potential as a sustainable alternative to animal proteins in food. Quinoa is currently of interest to researchers because of its nutritional and structural advantages. Quinoa is a rare plant food that provides all nine essential amino acids and is a complete source of protein [1]. This property is vital for the body’s repair and growth, as the body cannot synthesize essential amino acids and must obtain them from food [2]. In addition, quinoa itself is gluten-free, making it an ideal source of protein for those who suffer from celiac disease or follow a gluten-free diet. For example, a study by Bravi, Sileoni, and Marconi used quinoa as an ingredient in gluten-free biscuits [3]. Quinoa protein contains significant amounts of albumin and globulin. The primary storage proteins are 11S globulin and 2S albumin, accounting for approximately 37% and 35% of total protein, respectively [4]. The 11S globulin forms a hexamer consisting of six pairs of acidic (30–40 kDa) and alkaline (20–25 kDa) subunits linked by disulfide bonds. In contrast, 2S albumin is a dimer consisting of a small subunit (3–4 kDa) and a large subunit (8–9 kDa), also linked by disulfide bonds [5,6]. Current research indicates that despite its benefits, quinoa protein has limited gelling properties, low gel strength, and an insufficient water retention capacity. Research into quinoa albumin and quinoa globulin has not sufficiently explored their potential, hindering the full utilization of quinoa protein.

It has been observed that polysaccharides can significantly enhance the gel properties of proteins [7,8]. Xanthan gum exhibits unique rheological characteristics, excellent water solubility, and stability under thermal and acidic or basic conditions, making it a common choice as an emulsifier, stabilizer, and foaming agent in various industries such as food and pharmaceuticals [9,10]. Xanthan gum can markedly improve the gel properties of proteins through its multifaceted effects, including thickening, stabilization, and texture enhancement [11]. Our previous study also found that xanthan gum can improve the protein gel of quinoa [12].

Gelatinization plays an important role in food science, particularly in the development and improvement of plant-based foods. Recent studies have focused on improving the functional properties of gels to meet the demands of the food industry, such as improving texture, stability, and nutritional value. Research into plant proteins has mainly focused on soy and pea proteins [13,14]. However, legumes have anti-nutritional and allergenic properties that limit their use in foods [15]. In addition, the low solubility of several plant protein sources, such as rice and corn proteins, is a significant barrier to fully replacing animal proteins in stabilizing colloidal food systems [16].

The mechanisms underlying the formation of plant protein gels in response to heat stress encompass denaturation, aggregation, and gelation. These processes are contingent upon a few factors, including the composition of the protein, the pH, the ionic strength, and the presence of additional food components such as polysaccharides and polyphenols. Wang et al. demonstrated that the application of ultrasonic treatment results in the formation of a more ordered structure in mung bean protein gels. This treatment has been observed to enhance hydrophobic interactions and disulfide bonds, resulting in a denser and more uniform network [17]. Additionally, Tang et al. demonstrated that pH exerts a differential influence on the gel formation of pea protein and casein. Casein exhibits a proclivity to form a gel at a low pH, whereas chickpea gel formation takes place under neutral and alkaline conditions [18]. Liu et al. observed that curdlan reduces the number of free water molecules and the proportion of α-helices in soy protein isolate (SPI), resulting in a more stable SPI gel with a denser network [19]. While these studies emphasized the impact of external factors, few investigate the gel properties of albumin and globulin in plant proteins. Furthermore, research indicates that protein extraction conditions can influence protein aggregation and, subsequently, the gelatinization of proteins [20].

This study examined and compared gels made from ultrasound-treated quinoa protein, globulin, albumin, and polysaccharide complexes. The structural, rheological, texture, and functional properties of the gels were investigated to discuss the influence of protein components on quinoa protein–xanthan gum complex gels at different pH levels. This research not only addresses a gap in the literature but also has significant implications for enhancing the processing adaptability and utilization efficiency of quinoa protein in food science.

## 2. Results and Discussion

### 2.1. Effect of Protein Composition on Gel Structure at Different pH

#### 2.1.1. SDS-PAGE

**Figure 1:**
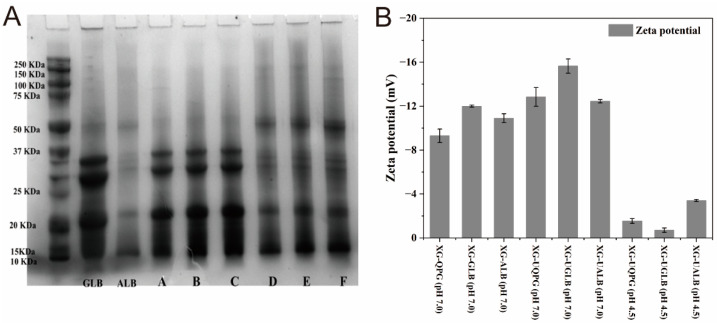
(**A**) (A–C) represent the SDS-PAGE strips of XG-GG (pH 7.0), XG-UGG (pH 7.0), and XG-UGG (pH 4.5), respectively. (D–F) represent XG-AG (pH 7.0), XG-UAG (pH 7.0), and XG-UAG (pH 4.5), respectively. (**B**) Effects of pH on the zeta potential of quinoa protein–xanthan gum complex gels.

Figure 1A illustrates the SDS-PAGE profiles for QPG, GG, and AG. A comparison of the globulin (GLB) and albumin (ALB) bands in Figure 1 demonstrates that XG does not result in the formation of new subunits in either globulin or albumin. The bands observed in XG-QPG (pH 7.0), XG-UQPG (pH 7.0), and XG-UQPG (pH 4.5) fall within the 10 kDa to 50 kDa range (Figure 1A). The primary subunit molecular weights for XG-GG (pH 7.0), XG-UGG (pH 7.0), and XG-UGG (pH 4.5) span from 20 to 25 kDa and up to 37 kDa. In contrast, XG-AG (pH 7.0), XG-UAG (pH 7.0), and XG-UAG (pH 4.5) exhibit a range of 10–50 kDa. These findings are consistent with those of Dakhili et al. [5], who identified peptides with molecular weights of 33–36 kDa as 11S acidic subunits, while those weighing 20–22 kDa correspond to 11S basic subunits. Brinegar and Goundan et al. [21] observed that all protein bands below 20 kDa correspond to albumin components. The available evidence suggests that the presence of 11S globulin is likely linked to gel quality. This is evidenced by the observation that XG-GG (pH 7.0), XG-UGG (pH 7.0), and XG-UGG (pH 4.5) exhibit a higher proportion of 11S globulin [22].

#### 2.1.2. Zeta Potential

The zeta potential value, as a quantitative indicator of the degree of mutual repulsion or attraction between particles in a system, is essential in assessing the stability of the system. As shown in Figure 1B, the zeta potential values of all samples are negative, indicating that the particle surfaces carry a negative charge. The ultrasonically treated samples (XG-UQPG, XG-UGG, and XG-UAG) show varying zeta potential values under the same pH conditions, with that of XG-UGG being significantly higher than those of the others, exceeding −15 mV. These results suggest that among the three protein components, globulin (GLB) is the most conducive to enhancing gel stability at pH 7.0. Furthermore, the results confirm that ultrasonic treatment not only alters the physical state of the samples but also significantly increases the negative charge on the particle surfaces, which helps enhance particle dispersion and stability.

Generally, the higher the absolute value of the zeta potential, the stronger the charge on the particle surface, and the greater the repulsive forces between the particles, which aids in maintaining the stability of suspensions or colloids [23]. However, this theory breaks down in solutions close to the isoelectric. As shown in Figure 1B, the zeta potential values of XG-UQPG (pH 4.5) and XG-UGG (pH 4.5) are close to zero at pH 4.5. However, these gels present good stability. This indicates that although the zeta potential has a certain effect on gel stability, it is not a decisive factor.

#### 2.1.3. FTIR

**Figure 2:**
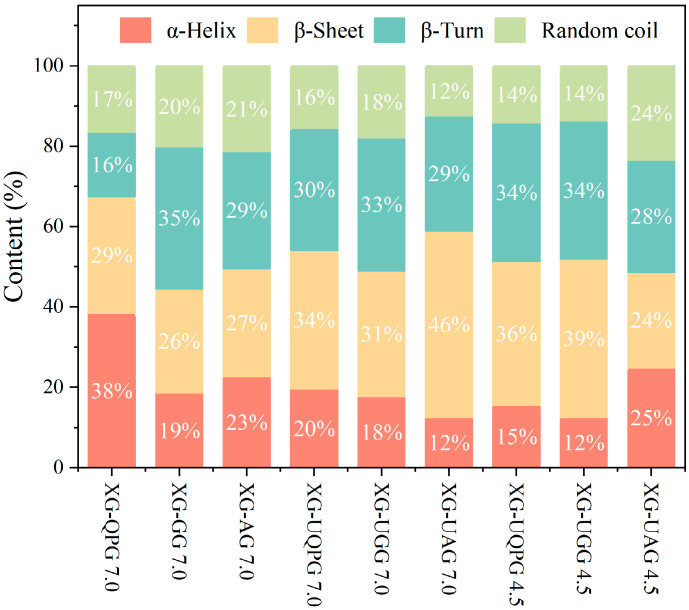
The influence of ultrasound on secondary structure.

The protein secondary structure of the samples was studied in detail by FTIR analysis. The conventional amide I band was used to fit the secondary structures of proteins in the gel samples, as shown in Figure 2. The β-sheet conformation is crucial for gel networks and protein–protein interactions, as this structure forms an ordered network of hydrogen bonds through its larger surface area, which is vital for the stability of gels. In contrast, random coils, being a disordered structure, do not generally favor the orderly formation of gel networks.

As shown in Figure 2, the secondary structures of proteins in XG-UQPG (pH 7.0), XG-UGG (pH 7.0), and XG-UAG (pH 7.0) exhibit an increased content of β-sheets and β-turns when compared with the proteins treated without ultrasound. The increase in β structure might be attributed to the mechanical effect of ultra-sonication, which could disrupt protein structure and convert α-helices into β structures. A similar result was observed by Zhang et al. [24]. Moreover, there are relevant studies that show the increase in content of β-folds and β-corners can improve gel strength [13]. On the other hand, under these conditions, XG-UGG and XG-UAG show higher proportions of β-sheets and β-turns compared to XG-UQPG, indicating a higher order of the composite gels is formed by GLB and ALB, consistent with the results involving disulfide bonds.

At pH 4.5, XG-UQPG and XG-UGG maintain a high level of secondary structure order, while XG-UAG shows a significant increase in the content of α-helices and random coils, reaching 25% and 24%, respectively, similar to the secondary structure distribution of ALB. These structural changes may be related to differences in protein components, especially ALB, which is primarily composed of 10 KDa small subunits. After ultrasonic treatment, the intermolecular structure of ALB is disrupted, exposing hydrophobic groups. However, due to the specific structural characteristics of ALB itself, these exposed groups do not lead to significant intermolecular rearrangement and aggregation. These findings provide a deeper understanding of protein structural changes under different pH conditions and ultrasonic treatments.

#### 2.1.4. Total Sulfhydryl and Disulfide Bonds

Table 1 provides a detailed listing of the total thiol, free thiol, and disulfide bond content in xanthan gum–quinoa protein composite gels. Initially, the ultrasonically treated samples at pH 7.0 demonstrate the following characteristics: XG-UQPG showed an increase in both total and free thiol contents; XG-UGG exhibits the highest total thiol and disulfide bond contents, but the lowest free thiol content; XG-UAG had a lower disulfide bond content compared to the untreated XG-AG. This suggests that at pH 7.0 and under ultrasonic conditions, GLB can form more disulfide bonds to maintain the stability of the gel structure, but ultrasonic treatment may inhibit the interaction between thiols in ALB.

Under pH 4.5 conditions, the free thiol content of XG-UQPG significantly increased, though the total thiol content decreased; XG-UGG had the highest free thiol content and still maintained a relatively high disulfide bond content; XG-UAG showed a very high free thiol content with a reduction in total thiol content compared to at pH 7.0. These results indicate that pH impacts the composite gels formed by different protein components differently. Under acidic conditions, thiols in QP are more prone to oxidation, leading to a reduction in total thiol content, while those in GLB are more stable. The increase in free thiols in XG-UAG suggests that acidic conditions promote the release of free thiols in ALB.

#### 2.1.5. SEM

**Figure 3:**
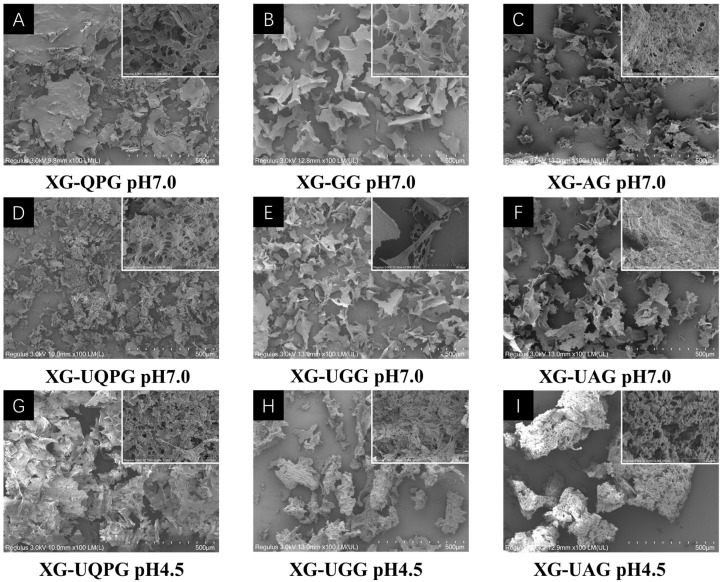
Effects of ultrasound and protein components on the microstructure of XG-QPG pH 7.0 (**A**), XG-GG pH 7.0 (**B**), XG-AG pH 7.0 (**C**), XG-UQPG pH 7.0 (**D**), XG-UGG pH 7.0 (**E**), XG-UAG pH 7.0 (**F**), XG-UQPG pH 4.5 (**G**), XG-UGG pH 4.5 (**H**), XG-UAG pH 4.5 (**I**).

Figure 3 illustrates the microstructure of xanthan gum (XG)–quinoa protein (QP) composite gels under an electron microscope. As depicted in Figure 3A–C, the structures of XG-UQPG and XG-UGG appear loose and with a more uniform pore size after sonication treatment. This phenomenon may arise due to the partial disruption of the rigid triple helix structures of QP, globulin (GLB), and albumin (ALB) into open triple helices or single helix chains during the thermal denaturation process. These structures intertwine with XG, forming a uniformly loose gel network and providing more hydrogen bonding sites for water molecules, thereby enhancing the gel’s water holding capacity (WHC).

At pH 4.5, the protein molecules’ surfaces in XG-UQPG (pH 4.5), XG-UGG (pH 4.5), and XG-UAG (pH 4.5) become rougher, with smaller pore sizes leading to less water molecule entrapment. The uniformity of protein gel is associated with the solubility of protein molecules, where higher solubility leads to a more uniform gel [25]. The main factors affecting the solubility of protein are pH and ionic strength [26]. At pH 4.5, the electrostatic charge of QP and GLB protein molecules is neutral, reducing the repulsive forces between protein molecules, facilitating aggregation, weakening the binding ability of water molecules, resulting in a rough gel structure and decreased WHC. Additionally, it is noteworthy that the findings of Klost [27] are consistent with ours, indicating that under low pH, the gel structure is rougher but stronger. Furthermore, when the pH approaches the isoelectric point of the protein, the net charge of the protein is neutral, minimizing the repulsive forces between molecules. At this point, hydrophobic interactions become dominant, promoting protein–protein cross-linking and gel formation [28]. This explains why the uniformity of the XG-UAG (pH 4.5) gel is significantly lower than that of XG-UQPG (pH 4.5) and XG-UGG (pH 4.5), leading to structural fragmentation. Specifically, the isoelectric point of ALB is 3.0, while that of QP and GLB is close to 4.5, resulting in differences in the interactions between ALB molecules and the other two proteins at pH 4.5.

### 2.2. Effect of Protein Composition on the Gel’s Rheological Properties at Different pH Levels

**Figure 4:**
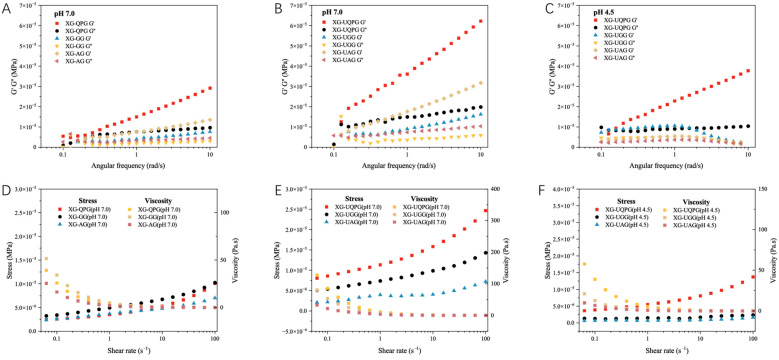
(**A**–**C**) Effects of ultrasound and protein components on viscoelastic properties of xanthan gum–quinoa protein complex gel solutions. (**D**–**F**) Effects of ultrasound and protein components on shear properties of xanthan gum–quinoa protein complex gel solutions.

Figure 4 represents the frequency sweeps and shear scans of the gel solutions. Figure 4A–C all show that for all gel solutions the storage modulus (G′) is greater than the loss modulus (G″), demonstrating the elastic characteristics of the gels. Among the groups, XG-UQPG (pH 7.0) and XG-UGG (pH 4.5) have the highest G′ values. Primarily, under pH 7.0 conditions, the G′ of XG-UQPG surpasses that of XG-UGG and XG-UAG. The thermal stability of proteins is closely related to their internal covalent cross-linking level, particularly the quantity of disulfide bonds. Compared to other protein intramolecular interactions, disulfide bonds require more energy to break during the gelation thermal processing process. With a lower disulfide bond content, XG-UQPG (pH 7.0) exhibits the highest G′. At pH 4.5, where the net charges of QP and GLB are neutral, minimizing intermolecular repulsion, favorable conditions exist for gel formation at relatively low protein concentrations. Specifically, XG-UGG (pH 4.5) demonstrates the highest G′, exhibiting a trend of initially increasing then decreasing. Studies by J.A.M. Berghout et al. [29] found that leguminous globulins from peas, soybeans, and beans possess varying amino acid compositions, sulfur contents, molecular weights, protein subunits, and surface hydrophobicities, all influencing their gel-forming abilities. Similarly, the rheological properties of thermally induced soybean protein gels are affected by the 7S/11S ratio in the protein, where gels rich in β-globulins and gels rich in glycine-containing proteins can achieve higher storage moduli [30]. At pH 4.5, partial insoluble aggregation occurs in globulins, leading to uneven gel solutions, thus demonstrating the trend of initial increase followed by decrease, and with a higher proportion of 11S proteins, XG-UGG (pH 4.5) exhibits the highest G′.

Figure 4D–F display the viscosity and shear stress of the gel as functions of the shear rate. With increasing shear rates, the shear stress of each sample increases while viscosity decreases correspondingly, demonstrating pseudo-plastic behavior. At pH 7.0, XG-UQPG (pH 7.0) exhibits the highest viscosity, possibly attributed to the improved dissolution and solution uniformity of GLB and ALB after sonication treatment. Moreover, the protein combines with xanthan gum to form a three-dimensional network structure [31], which limits the movement of water molecules, thus greatly enhancing the viscosity of the gel. However, at pH 4.5, due to reduced intermolecular repulsion between protein molecules, both stress viscosity and the required for strain decrease [32].

### 2.3. Effect of Protein Composition on the Gel’s Texture Properties at Different pH Levels

Table 2 provides the textural parameters of different gels, including hardness, springiness, chewiness, and resilience. Firstly, concerning gel hardness, it was observed that after sonication, the hardness of XG-UGG (pH 7.0) was the highest among the three samples, reaching 38 g. Furthermore, under pH 4.5 conditions, the hardness of XG-UGG (pH 4.5) and XG-UQPG (pH 4.5) (133 g and 130.68 g, respectively) was significantly higher than that of XG-UAG (pH 4.5), clearly demonstrating the influence of different protein components on the hardness of the composite gel.

Initially, at the same pH, ultrasound can promote an increase in gel strength. This could be attributed to the increase in β-sheets and β-turns in proteins induced by ultrasound, which could in turn enhance gel strength. A similar result was observed by Li et al. [13]. It is worth noting that although the content of disulfide bonds in GLB and ALB is relatively high, and the energy required to break these disulfide bonds differs during gel formation. Despite GLB having a higher disulfide bond content than ALB, the gel strength of GG is higher than that of AG. This may be attributed to GLB containing 11S and 7S subunits, which play crucial roles in gel formation. In the case of globular proteins, cross-linking occurs during heat-induced denaturation, leading to the formation of aggregates, the size of which increases with heating time until gel formation. The binding strength and structure of aggregates depend on the type and conditions of the proteins, but to form a network, aggregates need to have a self-similar structure on a large length scale. Wu et al. [22] found that a higher 11 S/7 S ratio results in a higher storage modulus for soy protein gels, mainly because peptides form larger and denser aggregates through hydrophobic bonds.

In terms of elasticity and resilience, GG performs the best among the three groups, followed by QPG and AG. This indicates that pure globulin can exert higher gelation, while albumin has a higher solubility but poor gelation due to its high non-gelatinous protein subunit composition.

Finally, concerning resilience, ultrasound treatment has little effect on gel resilience at pH 7.0 conditions. However, under acidic conditions (pH 4.5), the resilience of sonicated samples (XG-UQPG and XG-UGG) significantly increases. This change may be related to molecular-level alterations induced by ultrasound treatment, which could promote interactions between protein molecules, thereby enhancing gel resilience.

### 2.4. Effect of Protein Composition on the Functional Properties of Gels

#### 2.4.1. WHC and OHC

Table 3 presents the water holding capacity (WHC) and oil holding capacity (OHC) data for QPG, GG, and AG. From the tables, it can be observed that under pH 7.0 conditions, XG-UQPG (pH 7.0), XG-UGG (pH 7.0), and XG-UAG (pH 7.0) exhibit a relatively high WHC, with XG-UGG (pH 7.0) showing the most prominent WHC at 97.7%. This indicates that ultrasound has different effects on composite gels composed of different protein components. The principle behind this lies in the water in protein hydrogels being encapsulated within a spatially filled network formed by proteins connected through strong bonds [33]. Firstly, after ultrasound treatment, the protein structure unfolds, providing more binding sites for water molecules, enabling them to form hydrogen bonds with more water molecules, thus exhibiting a higher WHC. In addition, xanthan gum and protein interact with each other through electrostatic interaction and hydrogen bond to form a complex network structure, which also enhances the water holding capacity of the gels. This is also consistent with the results of the SEM images, where XG-UGG (pH 7.0) exhibits the most porous structure, hence it has the highest WHC.

At pH 4.5, it can be observed that only the WHC of XG-UGG (pH 4.5) remains at a relatively high level (89%), while the WHC of XG-UAG (pH 4.5) and XG-UQPG (pH 4.5) decreases. Under pH 4.5 conditions, proteins aggregate to form coarser protein chains, resulting in a denser gel structure, reduced pore size, and weakened water-binding capacity. XG-UGG (pH 4.5) also maintains a high WHC (89%), indicating that among the three protein components, the gel formed by GLB, XG-UGG (pH 4.5) can maintain a high WHC at a higher gel strength, making it a high-quality gel material. Lakemond et al. [32] studied the WHC of soy protein gels at alkaline and acidic pH values. They found that soy protein retains more water at pH 7.0 than at pH 3.8, similar to our findings.

Additionally, regarding the gel’s oil holding capacity, the OHC of all three gel types remains above 90%, with GG gel demonstrating the best oil holding capacity, indicating the potential of QPG, GLB, and ALB to form emulsifying gels, especially GLB.

#### 2.4.2. EAI and ESI

From Table 4, it is evident that different protein components have varying effects on the emulsifying properties of the gel.

Primarily, under pH 7.0 conditions, XG-UGLB exhibits the highest Emulsifying Activity Index (EAI), reaching 110.24 m²/g. Additionally, XG-UALB demonstrates favorable performance in terms of both the EAI and Emulsion Stability Index (ESI), with values of 90.89 m²/g and 81.45 min, respectively. Significant differences exist among gels composed of different protein components, with XG-UGG (pH 7.0) prepared using GLB showing the best emulsification performance.

However, when the pH is adjusted to 4.5, the EAI values of all samples decrease, with a more pronounced decline in ESI values. Particularly, the significant decrease in the ESI value of XG-UGG suggests a negative impact of pH reduction on emulsion stability. This effect may arise from the aggregation of GLB and QP molecules near their respective isoelectric points, leading to a reduction in the interaction between hydrophobic groups and oil.

In summary, it can be concluded that gels composed of GLB and ALB exhibit higher emulsifying activity, but the enhancement of emulsion stability does not always coincide with an increase in emulsifying activity. Additionally, pH variation significantly affects the performance of emulsifiers, with low pH environments being unfavorable for emulsifier stability. In the food industry, selecting suitable emulsifiers is crucial for developing food products with better texture, appearance, and taste. Considering that XG-UGG maintains the highest EAI and ESI at pH 7.0 and also sustains relatively high EAI and ESI values at pH 4.5, it is regarded as a promising gel material.

## 3. Conclusions

This study produced gels by xanthan gum and different QP, focusing on the effects of protein components and pH on gel properties. FTIR analysis demonstrated that the β-structure could promote the formation of a gel network. Moreover, AG and GG showed higher levels of disulfide bonds and free sulfhydryl groups, requiring more energy to break intermolecular sulfide bonds during heating. XG-UQPG exhibited superior rheological properties and gel strength compared to XG-UGG and XG-UAG. In addition, XG-UGG (pH 7.0) demonstrated the highest WHC and OHC, which was attributed to the disulfide bonds created in the proteins by ultrasound treatment, encouraging them to interact to form more uniform holes in gel that can hold more water/oil molecules. Conversely, at pH 4.5, the WHC of the gels were reduced due to the presence of rougher protein structures. These findings offer insights into enhancing the quality of quinoa protein gels.

## 4. Materials and Methods

### 4.1. Materials and Chemicals

The Gansu Academy of Agriculture Science provided the quinoa. Potassium bromide (KBr) and 5,5′-dithio-bis-nitrobenzoic acid (DTNB) were purchased from Aladdin Reagent Co. (Shanghai, China). Other chemicals were purchased from Sinopharm Chemical Reagent Co., Ltd. (Shanghai, China) and were of analytical grade.

### 4.2. Extraction of Protein

The quinoa protein (QP) isolate was derived using the methodology established in prior research [34]. Firstly, the peeled quinoa was ground and then passed through a 100-mesh sieve to achieve a fine consistency. Subsequently, the quinoa powder was degreased with n-hexane four times. Following the removal of fat, the quinoa powder was extracted using a 1:12 water ratio at 37 °C and a pH of 9 for a period of 3 h. The pH was then adjusted to 4.5 to precipitate the quinoa protein. The extraction and precipitation process was repeated three times. Subsequently, the pH was adjusted to 7.0, and the resulting protein was freeze-dried in preparation for subsequent experiments. The purity of the quinoa protein was found to be 85%.

Albumin (ALB) and globulin (GLB) fractions were extracted according to prior research [35] with some modification. The ABL was extracted with deionized water at 25 °C with continuous stirring at 120 rpm for 1 h, with a solid-to-liquid ratio of 1:10. The GBL was extracted with 1 M NaCl at 25 °C with continuous stirring at 120 rpm for 2 h, with a solid-to-liquid ratio of 1:10. Subsequently, the mixture was subjected to centrifugation at 4000× *g* for 10 min at 4 °C, enabling the recovery of the protein-rich supernatant. The pH of supernatants was adjusted to the isoelectric point of the protein (ALB: 3.0, GLB: 4.5) to obtain the precipitated protein. The precipitated proteins were collected and purified by dialysis. After that, the proteins were freeze-dried for subsequent experiments.

### 4.3. Ultrasonic Treatment of Protein

QP, ALB, and GLB were dissolved in deionized water to prepare a protein dispersion with a concentration of 6% (*w*/*v*). The pH of the protein dispersion was adjusted to 7.0 with 1 M NaOH and HCl, and it was continuously stirred and hydrated for 2 h. Then, the protein dispersions were treated by an ultrasonic processor (FS-1200 N, SHSONIC Instrument Co., Ltd., Shanghai, China). The 10 mm ultrasonic probe was inserted into the dispersing liquid, and the dispersing liquid was treated at 300 W and 20 kHz, and the ultrasonic density was controlled to reach 8 kJ/mL, respectively. The temperature of the sample in the centrifuge tube was controlled by an ice bath during the whole process of ultrasonic treatment. Ultrasonic density parameters can be calculated using the following formula:

(1) $$Ultrasonic\;density(kJ/mL)=\frac{W\times T}{V}$$

where *W* represents ultrasonic power, *T* represents ultrasonic time, and *V* represents the volume of the sample.

### 4.4. Preparation of Gels

Firstly, a 0.4% concentration solution of xanthan gum (XG) was prepared and stirred for 6 h before being left for overnight hydration for later use.

QP, ALB, and GLB were dissolved in deionized water to prepare a protein dispersion with a concentration of 6% (*w*/*v*). The pH of the protein dispersion was adjusted to 7.0 with 1 M NaOH and HCl, and it was continuously stirred for 3 h, then placed in a refrigerator overnight to allow for hydration. The XG solution was mixed 1:1 evenly with the protein solution (QP, ALB, and GLB) and heated at 90 °C for 40 min. Then, the mixed solutions were placed in a 4 °C refrigerator for 24 h to form the XG-QP gel (XG-QPG), XG-ALB gel (XG-AG), and XG-GLB gel (XG-GG).

The ultrasonically treated QP (UQP) solution, ultrasonically treated ALB (UA) solution, and ultrasonically treated GLB (UG) solution with a concentration of 6% (*w*/*v*) were also prepared. The pH of the protein solutions was adjusted to 7.0 or 4.5 with 1 M NaOH and HCl, and they were continuously stirred for 3 h. Then, they were placed in a refrigerator overnight to allow for hydration. The XG solution was mixed 1:1 evenly with the protein solution (UQP, UA, and UG) and heated at 90 °C for 40 min. Then, the mixed solutions were placed in a 4 °C refrigerator for 24 h to form the XG-UQP gel (XG-UQPG), XG-UA gel (XG-UAG), and XG-UG gel (XG-UGG) at different pH levels.

### 4.5. Sodium Dodecyl Sulfate-Polyacrylamide Gel Electrophoresis (SDS-PAGE)

SDS-PAGE was used to determine the molecular weight distribution as previously described [19]. Briefly, freeze-dried protein gels (1% *w*/*v*) were prepared in water and centrifuged for 10 min at 10,000× *g*. The supernatants were diluted with a 2× sample buffer and deionized water before applying the samples to Bio-Rad gels. Molecular weight markers (10–250 kDa) were obtained from Bio-Rad Laboratories Inc. The protein bands resolved upon electrophoresis were stained with Coomassie Blue Fast Staining Solution (Beyotime, Shanghai, China).

### 4.6. Zeta Potential

The samples were diluted to 0.2% (*w*/*v*) and transferred to a Malvern cuvette. Then, the zeta potential of the samples was measured using dynamic light scattering (Zetasizer Nano ZS, Malvern Instruments Co., Ltd., Worcestershire, UK). The duration for the measurement of zeta potential was 15 min, and three measurements were performed for each sample.

### 4.7. Fourier Transform Infrared Spectroscope (FTIR)

The FTIR analysis was conducted according to a previous study with some modification [36]. A total of 2 mg of freeze-dried protein gel was mixed with potassium bromide (KBr), ground, and then pressed into a pellet. A Nicolet IS-10 FTIR spectrometer (ThermoFisher Scientific, Marietta, OH, USA) was used to record the FTIR spectroscopy. The wavenumber ranged from 4000 cm^−1^ to 400 cm^−1^, at a 2 cm^−1^ resolution. Omnic V8.1 (ThermoFisher Scientific, USA) and PeakFit 4.12 (SeaSolve Software Inc., San Jose, CA, USA) were used to analyze the amide I band, and they determined the content of α-helix, β-sheet, turn, and unordered structure in the protein gels.

### 4.8. Total Sulfhydryl and Disulfide Bonds

The sulfhydryl and disulfide bond of proteins and gels were determined by the reference method [37]. For free sulfhydryl, the procedure was as follows: 0.02 m L of 4 mg/mL 5,5′-dithio-bis-nitrobenzoic acid (DTNB) and 2.5 mL of 8 mol/L urea solution (prepared with Tris-Gly) were added to 0.5 mL of 10 mg/m L sample and reacted for 25 min at 25 °C, and the absorbance values were measured at 412 nm. For total sulfhydryl, the procedure was as follows: We added 0.02 mL of β-hydrophobic ethanol and 1.0 mL of 10 mol/L urea solution (prepared by Tris-Gly) to 0.2 m L of 10 mg/m L sample, mixed well and let it react at 25 °C for 1 h. The absorbance was determined at 412 nm. After the reaction was continued for 1 h at 25 °C, the sample was centrifuged and washed twice. Then, the washed precipitate was dissolved with 3.0 mL of 8 mol/L urea solution (prepared with Tris-Gly), and 0.04 mL of DTNB was added. The reaction was conducted at 25 °C for 25 min, then measured for absorbance at 412 nm.

### 4.9. Scanning Electron Microscope (SEM)

Small gel fragments, with dimensions of approximately 5 × 5 × 5 mm, were excised from intact gel samples using a razor blade. Subsequently, the fragments were immersed in a 2.5% glutaraldehyde fixative for a period of 12 h. Subsequently, the sample crystals were subjected to rapid cold aging in liquid nitrogen at −196 °C, followed by freeze-drying. The desiccated samples were stored in a desiccator. The microstructures of the samples were examined using a SU8100 electron microscope, coated with gold, and accelerated at 3 kV.

### 4.10. Texture Property

The gel texture parameters were determined using a TA-TX2 plus texture analyzer (SMS, UK) as previously described with certain modifications [38]. A force–time curve was obtained at a crosshead speed of 0.50 mm/s for a 15 mm displacement, with pretest and posttest speeds set to 0.5 and 10 mm/s, respectively. Hardness, springiness, chewiness, gumminess, and resilience were calculated from the resulting TPA curves.

### 4.11. Rheological Property

The rheological properties of gels were analyzed using a rheometer (DHR-3, Waters, Milford, MA, USA). A parallel plate of 40 mm diameter was used for the experiment. The experiment was conducted in the frequency sweep mode with a frequency range of 0.1–10 Hz. The measurements were performed within the identified linear viscoelastic region to record the elastic modulus (G′ and G″) of gels. The viscosity of the gels was detected within shear rates of 0.1–100 s^−1^. All rheometer measurements were carried out at 25 °C.

The flow behavior index of the gels was calculated by fitting the experimental data to the Herschel–Bulkley model according to Equation (2) [39].

σ = σ_γ_ + K × γ^n^ (2)

where σ is the shear stress (Pa), σ_γ_ is the yield stress (Pa), K is the consistency coefficient (Pa·sn), γ is the shear rate (s^−1^), and n is the flow behavior index.

### 4.12. Water Holding Capacity (WHC) and Oil Holding Capacity (OHC)

We weighed a 0.5 g gel sample into a pre-weighed centrifuge tube, added 5 mL water, swirled for 30 s, left for 30 min, centrifuged, removed excess water, and weighed again. The *WHC* was calculated using Equation (3), given as follows:

(3) $$WHC=1-\frac{\left(\;w_{2}-w_{1}\right)}{w_{0}}\times 100$$

where *w*_0_ represents the initial weight of water in the gel (g) and *w_2_* − *_W1_* represents the weight of the expelled water (g).

We weighed a 0.5 g gel sample into a pre-weighed centrifuge tube, added 5 mL oil, swirled for 30 s, left for 30 min, centrifuged, removed excess oil, and weighed again. The calculation formula is as follows:

(4) $$OHC=1-\frac{\left(\;w_{2}-w_{1}\right)}{w_{0}}\times 100$$

where *w_0_* is the initial weight of oil in the gel (g) and *w_2_* − *w_1_* is the weight of the expelled oil (g).

### 4.13. Emulsifying Ability Index (EAI) and Emulsifying Stability Index (ESI)

*EAI* and *ESI* were determined using a previously established method [12] with slight modifications. Briefly, 0.05 mL of the emulsion was collected from the bottom of the beaker at 0 and 10 min and diluted with 0.1% *w*/*w* SDS. The optical density at 500 nm was measured using an SDPTOP UV−2400 spectrophotometer (Shimadzu, Guangzhou, China), with the SDS solution as a blank. The *EAI* and *ESI* values were calculated using Equations (5) and (6), given as follows:

(5) $$EAI\left(\frac{m^{2}}{g}\right)=\frac{2\times 2.303\times A^{0}\times D_{f}}{c\times \emptyset \times \left(1-\theta \times 10000\right)}$$

 

(6) $$ESI(min)=\frac{A^{0}}{A^{0}-A^{10}}\times 10$$

where *A*^0^/*A*^10^ represents the optical density of the diluted emulsion at 0 and 10 min, *D_f_* denotes the dilution multiple (100), *c* is the protein concentration of the gel solution (0.01 g/mL), and Φ represents the volume fraction of the oil in the emulsion (0.25).

### 4.14. Statistical Analysis

Experiments were conducted in triplicates, and the mean of the three data sets was calculated. Statistical analysis was conducted using SPSS 18.0 software. Differences were considered significant at *p* < 0.05.

## Figures and Tables

**Table 1:** Total sulfhydryl and disulfide bonds.

	Total Sulfhydryl Group	Free Sulfhydryl	Disulfide Bond
	(µmol/g)	(µmol/g)	(µmol/g)
XG-QPG (pH 7.0)	124.63 ± 2.81 ^e^	48.30 ± 0.60 ^d^	38.17 ± 1.70 ^f^
XG-GG (pH 7.0)	1257.01 ± 14.96 ^c^	84.10 ± 3.39 ^c^	586.46 ± 9.18 ^b^
XG-AG (pH 7.0)	930.51 ± 23.56 ^d^	144.11 ± 2.72 ^b^	393.20 ± 13.14 ^d^
XG-UQPG (pH7.0)	386.97 ± 17.16 ^f^	31.95 ± 0.30 ^de^	177.51 ± 8.43 ^e^
XG-UGG (pH 7.0)	1669.13 ± 47.15 ^a^	10.59 ± 0.69 ^e^	829.27 ± 23.23 ^a^
XG-UAG (pH 7.0)	970.97 ± 17.26 ^d^	19.78± 3.88 ^ef^	475.59 ± 10.57 ^c^
XG-UQPG (pH 4.5)	112.05 ± 0.36 ^e^	43.66 ± 6.47 ^d^	34.20 ± 3.42 ^f^
XG-UGG (pH 4.5)	1332.90 ± 12.42 ^b^	151.20 ± 4.74 ^b^	590.85 ± 3.84 ^b^
XG-UAG (pH 4.5)	943.41 ± 16.17 ^d^	229.41 ± 13.48 ^a^	357.00 ± 14.82 ^d^

^a–f^, The letters in a column indicate significant differences. Values are expressed as mean ± SD (*n* = 3).

**Table 2:** Effect of protein composition on gel texture property at different pH levels.

Samples	Hardness	Springiness	Chewiness	Resilience
	gf		gf	
XG-QPG (pH 7.0)	17.61 ± 0.76 ^d^	0.48 ± 0.01 ^d^	5.33 ± 0.53 ^d^	0.035 ± 0.000 ^de^
XG-GG (pH 7.0)	20.23 ± 0.89 ^d^	0.50 ± 0.01 ^d^	8.56 ± 1.10 ^c^	0.039 ± 0.001 ^d^
XG-AG (pH 7.0)	15.66 ± 0.45 ^d^	0.39 ± 0.01 ^e^	4.98 ± 0.24 ^c^	0.034 ± 0.001 ^de^
XG-UQPG (pH 7.0)	20.88 ± 1.94 ^d^	0.54 ± 0.02 ^c^	7.44 ± 0.34 ^c^	0.037 ± 0.002 ^d^
XG-UGG (pH 7.0)	38.07 ± 1.14 ^b^	0.56 ± 0.01 ^c^	15.48 ± 0.85 ^c^	0.061 ± 0.003 ^c^
XG-UAG (pH 7.0)	17.77 ± 1.03 ^d^	0.41 ± 0.01 ^e^	3.63 ± 0.31 ^c^	0.032 ± 0.001 ^e^
XG-UQPG (pH 4.5)	130.68 ± 11.7 ^a^	0.62 ± 0.02 ^b^	58.68 ± 8.63 ^a^	0.111 ± 0.003 ^a^
XG-UGG (pH 4.5)	133.44 ± 3.16 ^a^	0.66 ± 0.01 ^a^	41.34 ± 1.17 ^b^	0.113 ± 0.002 ^a^
XG-UAG (pH 4.5)	28.61 ± 1.45 ^c^	0.40 ± 0.01 ^e^	5.80 ± 0.75 ^c^	0.075 ± 0.002 ^b^

^a–e^, The letters in a column indicate significant differences. Values are expressed as mean. ± SD (*n* = 3).

**Table 3:** WHC and OHC of XG-QPG (pH7.0), XG-UQPG (pH7.0), and XG-UQPG (pH 4.5) at different protein concentrations.

	WHC	OHC
	%	%
XG-QPG (pH 7.0)	80.38 ± 0.39 ^d^	95.05 ± 0.13 ^b^
XG-GG (pH 7.0)	81.46 ± 0.60 ^cd^	97.64 ± 0.61 ^ab^
XG-AG (pH 7.0)	81.05 ± 0.12 ^d^	97.25 ± 0.40 ^ab^
XG-UQPG (pH 7.0)	90.48 ± 0.37 ^b^	98.98 ± 0.03 ^a^
XG-UGG (pH 7.0)	97.70 ± 0.76 ^a^	99.26 ± 0.54 ^a^
XG-UAG (pH 7.0)	81.80 ± 0.43 ^cd^	97.95 ± 1.12 ^ab^
XG-UQPG (pH 4.5)	83.70 ± 1.55 ^c^	96.68 ± 1.11 ^ab^
XG-UGG (pH 4.5)	89.64 ± 1.12 ^b^	98.38 ± 1.55 ^a^
XG-UAG (pH 4.5)	71.29 ± 0.17 ^e^	97.89 ± 0.53 ^ab^

^a–e^, The letters in a column indicate significant differences. Values are expressed as mean. ± SD (*n* = 3).

**Table 4:** Effect of protein components on the EAI and ESI of xanthan gum–quinoa protein complex gels.

	Emulsifying Property m^2^/g	Emulsion Stability min
XG-QPG (pH 7.0)	19.65 ± 0.01 ^f^	24.76 ± 1.90 ^d^
XG-GG (pH 7.0)	93.96 ± 0.61 ^b^	20.61 ± 2.73 ^de^
XG-AG (pH 7.0)	90.28 ± 0.61 ^bc^	51.72 ± 2.72 ^bc^
XG-UQPG (pH 7.0)	88.28 ± 1.22 ^c^	80.05 ± 3.34 ^a^
XG-UGG (pH 7.0)	110.24 ± 3.38 ^a^	48.51 ± 1.49 ^c^
XG-UAG (pH 7.0)	90.89 ± 0.61 ^bc^	81.45 ± 4.02 ^a^
XG-UQPG (pH 4.5)	40.47 ± 1.53 ^e^	59.32 ± 2.27 ^b^
XG-UGG (pH 4.5)	81.99 ± 2.15 ^d^	13.69 ± 0.29 ^e^
XG-UAG (pH 4.5)	81.68 ± 0.61 ^d^	14.95 ± 0.17 ^e^

^a–e^, The letters in a column indicate significant differences. Values are expressed as mean. ± SD (*n* = 3).

## Data Availability

The original contributions presented in the study are included in the article; further inquiries can be directed to the corresponding author.
